# Improved hybrid *de novo* genome assembly and annotation of African wild rice, *Oryza longistaminata*, from Illumina and PacBio sequencing reads

**DOI:** 10.1002/tpg2.20001

**Published:** 2020-03-20

**Authors:** Wei Li, Kui Li, Qun‐jie Zhang, Ting Zhu, Yun Zhang, Cong Shi, Yun‐long Liu, En‐hua Xia, Jian‐jun Jiang, Chao Shi, Li‐ping Zhang, Hui Huang, Yan Tong, Yuan Liu, Dan Zhang, Yuan Zhao, Wen‐kai Jiang, You‐jie Zhao, Shu‐yan Mao, Jun‐ying Jiao, Ping‐zhen Xu, Li‐li Yang, Guo‐ying Yin, Li‐zhi Gao

**Affiliations:** ^1^ Inst. of Genomics and Bioinformatics South China Agricultural Univ. Guangzhou 510642 China; ^2^ Plant Germplasm and Genomics Center Germplasm Bank of Wild Species in Southwestern China, Kunming Inst. of Botany, Chinese Acad. of Sciences Kunming 650204 China; ^3^ College of Life Science Liaoning Normal Univ. Dalian 116081 China; ^4^ Univ. of the Chinese Acad. of Sciences Beijing 100039 China; ^5^ Yunnan Agricultural University Kunming 650201 China

## Abstract

African wild rice *Oryza longistaminata*, one of the eight AA‐ genome species in the genus *Oryza*, possesses highly valued traits, such as the rhizomatousness for perennial rice breeding, strong tolerance to biotic and abiotic stresses, and high biomass production on poor soils. To obtain the high‐quality reference genome for *O. longistaminata* we employed a hybrid assembly approach through incorporating Illumina and PacBio sequencing datasets. The final genome assembly comprised only 107 scaffolds and was approximately ∼363.5 Mb, representing ∼92.7% of the estimated African wild rice genome (∼392 Mb). The N50 lengths of the assembled contigs and scaffolds were ∼46.49 Kb and ∼6.83 Mb, indicating ∼3.72‐fold and ∼18.8‐fold improvement in length compared to the earlier released assembly (∼12.5 Kb and 364 Kb, respectively). Aided with Hi‐C data and syntenic relationship with *O. sativa*, these assembled scaffolds were anchored into 12 pseudo‐chromosomes. Genome annotation and comparative genomic analysis reveal that lineage‐specific expansion of gene families that respond to biotic‐ and abiotic stresses are of great potential for mining novel alleles to overcome major diseases and abiotic adaptation in rice breeding programs. This reference genome of African wild rice will greatly enlarge the existing database of rice genome resources and unquestionably form a solid base to understand genomic basis underlying highly valued phenotypic traits and search for novel gene sources in *O. longistaminata* for the future rice breeding programs.

**Abbreviations**
BUSCO: benchmarking universal single‐copy orthologs
BWA: burrows wheeler aligner
EVM: EVidenceModeler
GO: gene ontology
LTR: long terminal repeat
ncRNA: non‐coding RNA
ORF: open reading frame
PacBio: Pacific Biosciences
PASA: Program to Assemble Spliced Alignments
SMRT: Single Molecule Real Time
SSR: simple sequence repeat
TE: transposable element

## 1 INTRODUCTION

*Oryza longistaminata* A. Chev. & Roehr. is among the eight AA‐ genome species in the genus *Oryza*. This wild rice species, which is native to Africa, owns highly prized traits for genetic improvement of cultivated rice, including well‐made rhizomes for perennial rice breeding (Glover et al., 2010), strong tolerance to biotic and abiotic stresses (Brar & Khush, 1997, Tanksley & McCouch, 1997, Xiao et al., 1998), and high biomass production on poor soils. Decoding the *O. longistaminata* genome is undoubtedly a key to uncover molecular mechanisms that determine these remarkable agronomic traits and enhance rice genetic improvement. We recently sequenced the five AA‐ genome rice species, and obtained the first insights into the rice genome adaptation (Zhang et al., 2014). As a result of the nature of inherent self‐incompatibility in *O. longistaminata*, however, it remains a great challenge to decipher this genome because of its relatively high heterozygosity of 1.05% estimated by 17 *k*‐mer distribution (Figure S1).

A high‐quality *O. longistaminata* genome assembly is fundamental to mine rich source of novel alleles that are of great importance for breeding new rice cultivars in the future. Based on a large quantity of combined sequencing data (∼396 × Illumina short reads and ∼5.9 × Roche GS FLX reads), Zhang et al. (Zhang et al., 2015) reported a draft genome assembly of *O. longistaminata* with a short (12.5 Kb) contig N50 length. The disadvantage of low‐quality genome assembly makes it difficult to gain worthwhile information of genomic variation, functional genomic studies as well as efficient germplasm utilization. Here we report a high‐quality genome assembly as well as large transcriptome datasets for *O. longistaminata* using Illumina and PacBio sequencing platforms.

## 2 MATERIALS AND METHODS

### 2.1 Plant materials

An individual plant of *O. longistaminata* (IRGC 81967) originally from Botswana was introduced and grown in the greenhouse of Kunming Institute of Botany, Chinese Academy of Sciences. The collected leaf samples were immediately frozen in liquid nitrogen and stored at −80°C in the laboratory.

### 2.2 DNA extraction, library construction and sequencing

The nuclear DNA was isolated from young leaves using a modified CTAB method. The extracted DNA was examined using a NanoDrop D‐1000 spectrophotometer (NanoDrop Technologies, Wilmington, DE) and electrophoresis on a 0.8% agarose gel, respectively. According to the Illumina's protocol, three short‐insert (300 bp, 360 bp and 500 bp) paired‐end and five long‐insert (2 Kb, 4 Kb, 5 Kb, 6 Kb and 8 Kb) mate‐pair genomic DNA libraries were constructed and sequenced using Hiseq2000 platform. 20 μg of high‐quality DNA was used to construct 20‐Kb SMRT Bell library. The library was then sequenced on the PacBio RSII platform with P6‐C4 chemistry combination.

### 2.3 Estimation of the genome size

Two orthogonal methods were used to estimate the genome size of *O. longistaminata*, including *k*‐mer frequency distribution and flow cytometric analyses. We generated the 17‐mer occurrence distribution of sequencing reads from short libraries (≤500 bp) using Jellyfish (version 2.1.3) (Marcais & Kingsford, 2011). The genome size and heterozygosity were then estimated using GCE version 1.0 (Liu, Shi, Yuan, Hu, & Wei, 2013) based on the *k*‐mer counting.

The genome size of the *O. longistaminata* was further assessed by flow cytometry analysis. Approximately 50 mg of fresh leaves was used for sample preparation according to Huang et al. (Huang, Tong, Zhang, & Gao, 2013) using Otto's lysis buffer (Loureiro, Rodriguez, Dolezel, & Santos, 2006). A BD FACSCalibur (USA) flow cytometer was used to estimate the nuclear DNA amount. The Cellquest software (version 5.1) was employed to analyze the mean ratio of *G*_0/1_ (Nuclei) peak and the internal standard. We selected the rice cultivar *Nipponbare* as inner standard with an estimated genome size of 389 Mb (IRGSP., 2005), and estimated the genome size of *O. longistaminata* based on the linear relationship of 2C peaks.

### 2.4 Hybrid *de novo* genome assembly and quality assessment

Low‐quality raw reads were trimmed using Trimmomatic (version 0.33) (Bolger, Lohse, & Usadel, 2014). ALLPATHS‐LG (Gnerre et al., 2011) was then used to assemble the *O. longistaminata* genome. Gaps between scaffolds were then filled with GapCloser (version 1.12) (Luo et al., 2012) based on all pair‐end reads. Potential bacterial‐derived scaffolds were removed by Blast searches against bacterial genomes with an identity of ≥95% and E‐value ≤1 × 10^‐5^. Based on the above‐generated *de novo* genome assembly using Illumina sequencing reads, we used ∼7.28 Gb (18.57 ×) PacBio RS reads longer than 500 bp in the hybrid assembly using the PBJelly 2 (English et al., 2012) with default parameters. Finally, Lachesis (Burton et al., 2013) was used to link the scaffolds into pseudo‐chromosomes with default parameters.

Three approaches were used to evaluate the assembly quality of the *O. longistaminata* genome. First, we mapped all high‐quality reads (∼21.6 Gb) to the assembled genome using BWA (version 0.7.15) (Li & Durbin, 2009). Second, the completeness of the assembly was assessed with BUSCO (Simao, Waterhouse, Ioannidis, Kriventseva, & Zdobnov, 2015) collected from Embryophyta lineage. Finally, RNA sequencing reads generated in this study were assembled into transcripts using Trinity (version 2.0.6) (Grabherr et al., 2011), which were then aligned back to our genome assembly using GMAP (Wu & Watanabe, 2005) at 90% coverage and 90% identity thresholds.

### 2.5 RNA extraction and transcriptome sequencing

Total RNA was isolated from the four tissues of *O. longistaminata*, including 30‐d‐roots, 30‐d‐shoots, panicles at booting stage and flag leaves at booting stage, using a Water Saturated Phenol method (Zhang et al., 2014). Following the Illumina's recommendations, RNA sequencing libraries were constructed and paired‐end sequenced on the Illumina Hiseq2000 platform, generating ∼26.73 Gb of RNA sequencing (RNA‐Seq) data.

**Core Ideas**

- We assembled a reference genome of *O. longistaminata* using a hybrid assembly approach.
- The N50 lengths of contigs and scaffolds reached to ∼46.49 Kb and ∼6.83 Mb, respectively.
- We anchored the 107 assembled scaffolds into 12 pseudo‐chromosomes by Hi‐C data.
- We detected the expansion of gene families involved in biotic‐ and abiotic stresses.
- We generated a large number of genetic markers to assist rice‐breeding programs.

### 2.6 Genome annotation

Repetitive sequences of the *O. longistaminata* genome assembly were masked prior to gene prediction. A combined strategy was adopted to predict the protein‐coding genes of *O. longistaminata*. Augustus (version 3.0.3) (Stanke, Steinkamp, Waack, & Morgenstern, 2004), GlimmerHMM (version 3.0.3) (Majoros, Pertea, & Salzberg, 2004) and GeneMarkHMM (version 3.47) (Lukashin & Borodovsky, 1998) were used to detect the hypothetical gene‐coding regions in the *O. longistaminata* genome. The protein sequences from *Oryza*, *Zea mays*, *Sorghum bicolor*, and *Brachypodium distachyon* were aligned to the *O. longistaminata* genome assembly using GenBlastA (version 1.0.1) (She, Chu, Wang, Pei, & Chen, 2009) and further refined using GeneWise (version 2.2.0) (Birney, Clamp, & Durbin, 2004). To further aid the gene annotation Illumina RNA‐seq reads (∼26.73 Gb) were first assembled into transcripts using Trinity (version 2.0.6) (Grabherr et al., 2011), and then aligned to the genome assembly using PASA (Program to Assemble Spliced Alignments) (Haas et al., 2003) to determine the potential gene structures. EVidenceModeler (EVM) (Haas et al., 2008) was used to combine all predicted results from *ab initio*, protein and EST evidences into consensus gene predictions. We filtered out gene models with their peptide lengths ≤50aa and/or harboring stop codons to obtain the final predicted genes. The functional motifs and domains were determined using InterProScan (version 5.3) (Jones et al., 2014). PFAM domains and GO terms were directly retrieved from the corresponding InterPro entries.

TEs were annotated integrating RepeatMasker (www.repeatmasker.org), LTR_STRUC (McCarthy & McDonald, 2003), RECON (Bao & Eddy, 2002), and LTR_Finder (Xu & Wang, 2007). All intact LTR retrotransposons generated by LTR_STRUC were classified into Ty1‐*copia*, Ty3‐*gypsy*, and unclassified superfamilies according to the order of ORFs using PFAM (Punta et al., 2012). The 5′‐ and 3′‐LTR were aligned using ClustalW (Larkin et al., 2007) and the *K*‐value (the average number of substitutions per aligned site) was calculated by PAML (Yang, 2007). The insertion times (*T*) were calculated using the formula: *T* = *K*/2*r*, where *r* represents the substitution rate, which is 1.3 × 10^−8^ per site per year (Baucom, Estill, Leebens‐Mack, & Bennetzen, 2009, Vitte, Panaud, & Quesneville, 2007). SSRs in the *O. longistaminata* genome were identified using microsatellite identification tool (MISA) (Thiel, Michalek, Varshney, & Graner, 2003).

### 2.7 Gene family evolution analysis

To avoid potential contamination introduced by TE‐related proteins, the predicted genes were blasted against the MSU *Oryza* Repeat Database. OrthoMCL (Li, Stoeckert, & Roos, 2003) was used to obtain paralogous and orthologous gene families with the finished genomes: *O. sativa*, *O. longistaminata*, *B. distachyon*, and *Arabidopsis thaliana*. Then, we used RAxML package (Stamatakis, 2006) to construct a phylogenetic tree of single‐copy gene families. Subsequently, we used CAFÉ (version 2.2) (De Bie, Cristianini, Demuth, & Hahn, 2006) to detect the expansion and contraction of gene families. To further examine the evolutionary relationships in the genus *Oryza*, MCScan (Haibao et al., 2008) was used to construct genome collinearity blocks. The predicted genes for the two *Oryza* species were used to all‐vs‐all comparisons.

### 2.8 Data availability

The raw sequence data and genome assembly reported in this paper have been deposited in BIG Data Center (Nucleic Acids Res 2018) and NCBI (National Center for Biotechnology Information) Data Center under accession numbers PRJCA000900 and PRJNA545798, respectively.

## 3 RESULTS AND DISCUSSION

### 3.1 Genome sequencing

The nuclear genome of *O. longistaminata* was sequenced using whole‐genome shotgun sequencing (WGS). Based on the 17‐mer frequency distribution of sequencing reads, the genome size of *O. longistaminata* was estimated to be ∼392 Mb, and flow cytometry analysis further showed that the *O. longistaminata* genome was approximately estimated to be ∼363 Mb. We obtained a total of 166.25 Gb of clean Illumina data, representing ∼424‐fold coverage (Supplemental Table S1). Then we performed single‐molecule long read sequencing on the PacBio platform. This process produced ∼7.28 Gb data, consisting of 873,994 reads with a length of 8,324 bp on average (Supplemental Figure S2; Supplemental Table S1).

### 3.2 Hybrid *de novo* genome assembly and quality assessment

**FIGURE 1:**
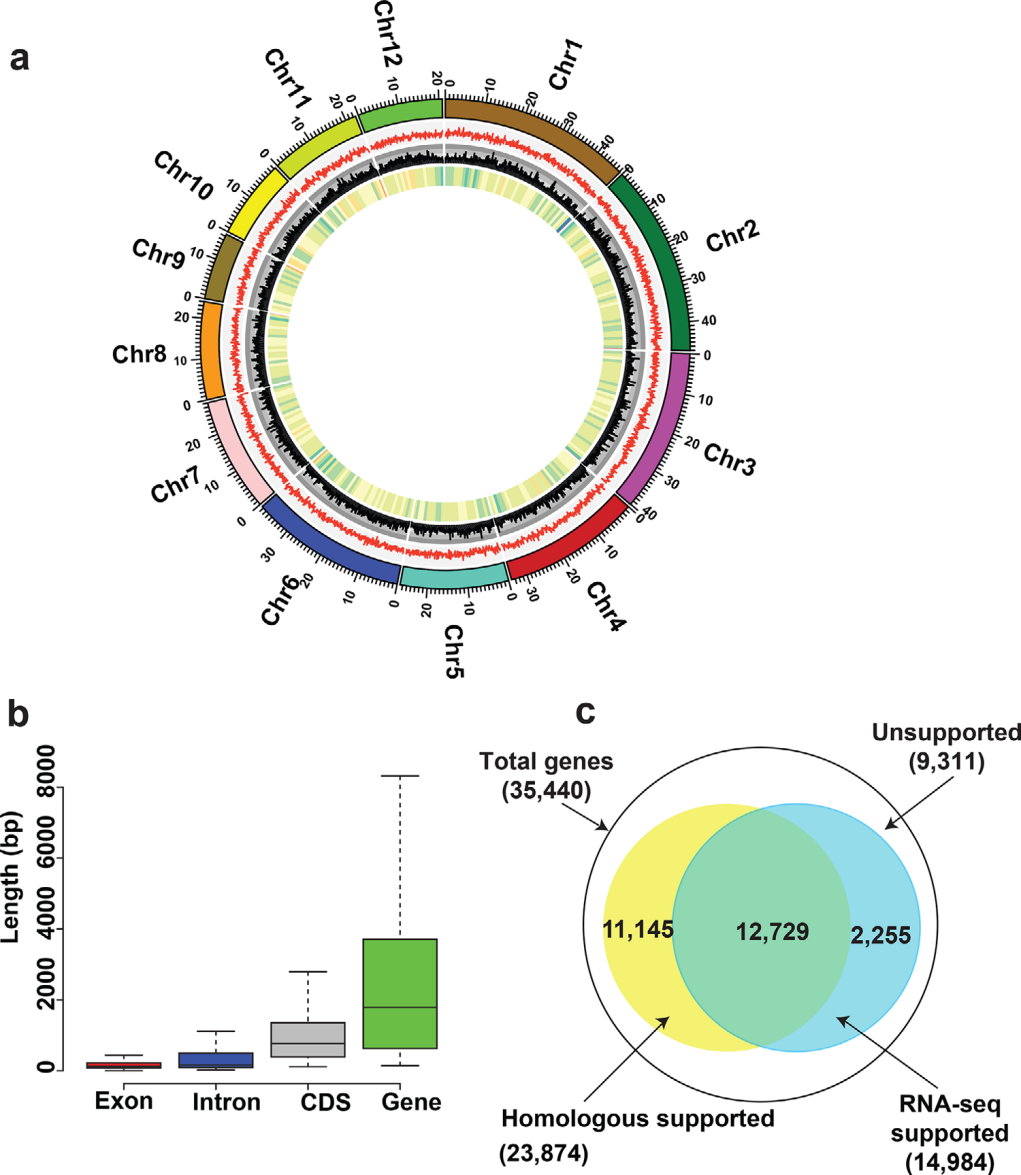
Characteristics of the *O. longistaminata* genome. (a) Tracks from outside to inside are the 12 chromosomes of *O. longistaminata*, GC content, transposable element (TE) density, and SSR density; (b) Parameters of the predicted genes; (c) Validation of gene models

Heterozygosity is a challenging problem that complicates to assemble genomes, resulting in highly fragmented genome assemblies (Michael & VanBuren, 2015). A hybrid assembly method was employed to confront high heterozygosity in the *O. longistaminata* genome (Supplemental Figure S3). Firstly, the genome of *O. longistaminata* was assembled using ALLPATHS‐LG (Gnerre et al., 2011), yielding an initial *O. longistaminata* genome assembly of ∼331 Mb. The N50 lengths of the assembled contigs and scaffolds were ∼17.06 Kb and ∼1.11 Mb, respectively (Supplemental Table S2). Secondly, the SMRT reads were further added, yielding a genome assembly of ∼371.3 Mb, representing ∼94.7% of the estimated genome size (∼392 Mb) (Supplemental Table S3). The N50 lengths of the assembled contigs and scaffolds increased to ∼45.65 Kb and ∼1.24 Mb, respectively (Supplemental Table S2). A total of 433 scaffolds larger than 100 Kb in length occupied 97.50% of the genome assembly (Figure S4a; Figure S4b). Aided with ∼116 Gb (296 × genome coverage) Hi‐C data we further linked these scaffolds using Lachesis (Burton et al., 2013) with default parameters. This produced only 107 scaffolds with a total length of the final genome assembly of ∼363.5 Mb, representing ∼92.7% of the estimated genome size for the African wild rice (Table 1; Supplemental Table S4). The N50 lengths of the assembled contigs and scaffolds increased to ∼46.49 Kb and ∼6.83 Mb, respectively (Table 1; Supplemental Table S4). The contig N50 and scaffold N50 sizes represent ∼3.72‐fold and ∼18.8‐fold improvement compared with previously reported ∼12.5 Kb and 364 Kb (Zhang et al., 2015), respectively. It is apparent that the genome assembly were largely improved after adding long SMRT reads, making it feasible to accurately predict protein‐coding genes and sufficiently annotate repeat sequences. We finally assembled scaffolds of *O. longistaminata* into 12 pseudo‐chromosomes (Figure 1a; Supplemental Table S5) based on Hi‐C data and syntenic relationships with *O. sativa* ssp. *japonica* cv. *Nipponbare* (MSU 7.0). The assembled genome was referred to as Oryza_longistaminata_v2.0.

**Table 1:** Summary of genome assembly and annotation of *O. longistaminata*

	Illumina + Roche^a^	Illumina + PacBio + HiC
Genome Assembly		
Data for initial assembly (×)	402.44	442.68
Estimated genome size (Mb)	350.97	392.00
Assembled sequence length (Mb)	347.79	363.52
Scaffold N50 (bp)	363,997	6,832,378
Contig N50 (bp)	12,515	46,493
Percentage of gap sequences (%)	N/A	2.30
Genome Annotation		
Number of predicted genes	32,502	35,440
Average gene length (bp)	2,836	2,990
tRNAs	720	755
rRNAs	34	4
snoRNAs	N/A	274
snRNAs	669	143
miRNAs	3,954	299
Percentage of repeat sequences (%)	48.73	33.09

a Zhang et al., 2015.

For the validation of genome assembly, we first mapped all high‐quality reads (∼21.6 Gb) to the assembled genome sequence, and obtained an average mapping rate of 86.48% (Supplemental Table S6). We then checked the genome assembly with benchmarking universal single‐copy orthologs (BUSCO) (Simao et al., 2015). Our results displayed 89.86% (1,294 of 1,440) complete BUSCOs in the Embryophyta lineage (Supplemental Table S6). Finally, the transcripts of *O. longistaminata* were mapped to the assembled genome. Our results showed that a total of 143,778 transcripts could be successfully aligned to the genome with a mapping rate of 60.58% (Supplemental Table S6).

### 3.3 Transcriptome sequencing and *de novo* assembly

We performed transcriptome sequencing to improve the quality of the *O. longistaminata* genome annotation. A total of ∼26.73 Gb of RNA sequencing (RNA‐Seq) data was generated, representing major tissue types and developmental stages, including 30‐d‐shoots, 30‐d‐roots, panicles at booting stage and flag leaves at booting stage (Supplemental Table S7). The clean data was assembled using Trinity (version r20140717) (Grabherr et al., 2011) and only the longest contigs per component were counted, yielding a total of 111,105 transcripts with a N50 value of 1,064 bp (Supplemental Table S8).

### 3.4 Genome annotation

A total of 35,440 protein‐coding genes were predicted in the *O. longistaminata* genome assembly (Supplemental Table S9; Figure 1b). We aligned the annotated protein‐coding genes of *O. sativa* and RNA‐seq data of *O. longistaminata* generated in this study to assess the quality of gene prediction. Our results showed that 73.7% of the predicted gene models was supported by transcript or protein evidences (Figure 1c), demonstrating a high accuracy of the gene prediction of the *O. longistaminata* genome.

Non‐coding RNA (ncRNA) genes were predicted using *ab initio* and/or homology search methods (Zhang et al., 2014). We identified 755 transfer RNA (tRNA) genes, 4 ribosomal RNA (rRNA) genes, 274 small nucleolar RNA (snoRNA) genes, 143 small nuclear RNA (snRNA) genes, and 299 microRNA (miRNA) genes (Table 1; Supplemental Table S10).

**FIGURE 2:**
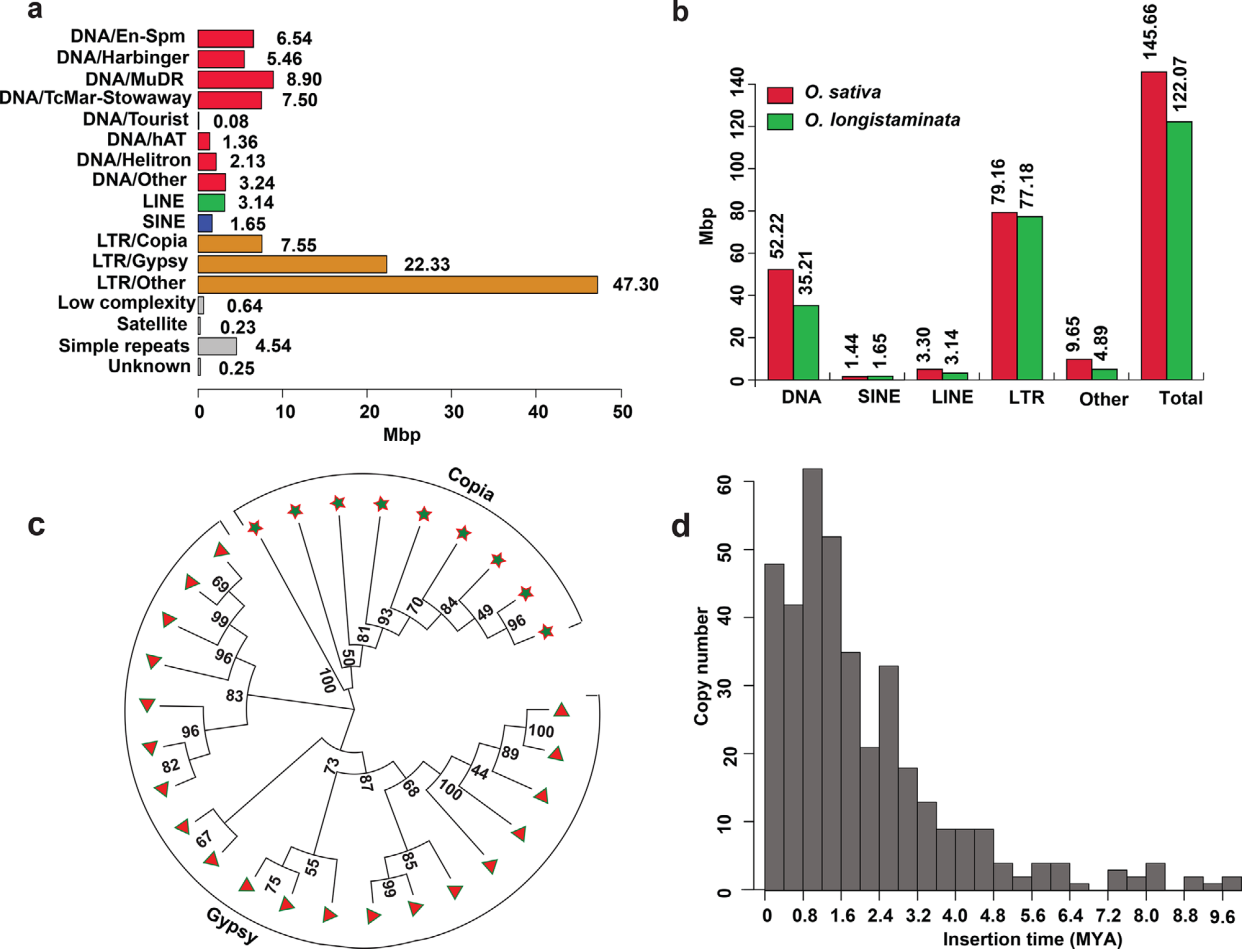
Annotation of transposable elements. (a) Repeat content of the *O. longistaminata* genome assembly. (b) Comparison of repeat sequences between *O. longistaminata* and *O. sativa*. (c) Phylogenetic tree of *Gypsy* and *Copia* superfamilies constructed from the RT domains without premature termination codon. (d) The insertion times for intact LTR retrotransposons in the *O. longistaminata* genome

The annotation of repeat sequences showed that approximately 33.09% of the *O. longistaminata* genome consisted of transposable elements (TEs) (Supplemental Table S11; Figure 2a), which is relatively lower than in the *Nipponbare* genome (∼39.40%) (Figure 2b). Long terminal repeat (LTR) retrotransposons occupied ∼20.79% of the *O. longistaminata* genome (Supplemental Table S11). Ty3‐g*ypsy* superfamilies appeared more abundant (6.01%) than Ty1‐c*opia* superfamilies (2.03%) (Supplemental Table S11; Figure 2c). Molecular dating suggested that nearly 60% of LTR retrotransposons came to the *O. longistaminata* genome within the last 2 MYA (Figure 2d), which is consistent with earlier findings in the rice genome (Gao, McCarthy, Ganko, & McDonald, 2004; Tian et al., 2009).

A total of 197,053 simple sequence repeats (SSRs) were identified in the *O. longistaminata* genome, and the frequency of occurrence was estimated to be one SSR every 1.88 kb (Supplemental Table S12). Mono‐, di‐, tri‐, tetra‐, penta‐, and hexa‐mer accounted for 13.93%, 13.94%, 34.31%, 25.81%, 7.71%, and 4.30% of the identified SSRs, respectively (Supplemental Table S12). These identified SSRs can be used as valuable genetic markers for the germplasm characterization and utilization of *O. longistaminata*.

### 3.5 Rice gene family evolution

**FIGURE 3:**
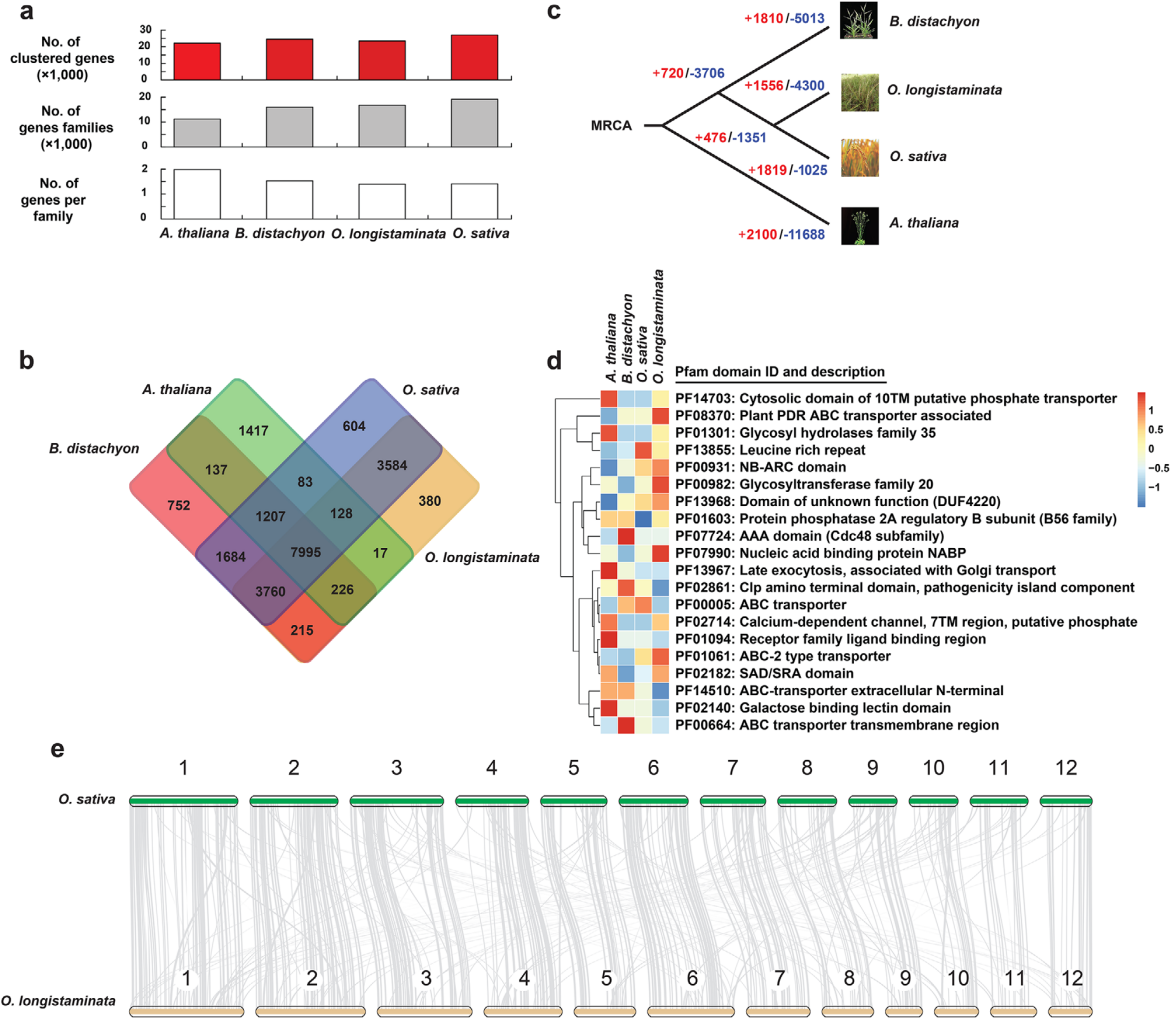
Comparative genomic analysis of rice, *B. distachyon*, *A. thaliana*, *O. longistaminata*. (a) Number of clustered genes, gene families and gene family sizes of the four sequenced plant genomes. (b) Venn diagram showing the distribution of shared and specific gene families among the four plant species. (c) Gene family expansion (red) or contraction (blue) among the four plant species. (d) Heat map of Pfam domains with significantly biased distribution. Only the top 20 significant categories were shown. (e) Collinear blocks between rice and *O. longistaminata*. Each line connects a pair of orthologous genes between the two genomes

The predicted protein‐coding genes of *O. longistaminata* were compared with those from rice (IRGSP., 2005), *A. thaliana* (The Arabidopsis Genome Initiative, 2000), and *B. distachyon* (The International Brachypodium Initiative, 2010). A total of 134,448 genes were clustered into 22,189 gene families (Figure 3a). Of these, 7,995 gene families, which contained 50,443 genes, were common to all four plant species, representing ancestral gene families (Figure 3b). There were 380 gene families containing 861 genes unique to *O. longistaminata*, of which some were potentially related to important biological processes (Figure 3b). Further functional annotation based on PFAM terms revealed that a total of 380 lineage‐specific gene families were enriched with genes associated with wall‐associated receptor kinase galacturonan‐binding (PF13947), cathepsin propeptide inhibitor domain (PF08246) and pectinesterase (PF01095) (Supplemental Figure S5). Our data analysis revealed that a total of 1,556 and 4,300 gene families underwent significant expansion and contraction, respectively (Figure 3c). PFAM annotation showed that the defense genes were highly enriched in the expanded gene families, such as NB‐ARC domain (PF00931) and leucine rich repeat (PF00560) (Figure 3d). This is consistent with earlier findings that *O. longistaminata* exhibits a high resistance to insect pests and diseases (Song et al., 1995), suggesting a great potential for mining novel alleles to overcome major diseases and abiotic adaptation in rice breeding program. A total of 1,200 syntenic blocks covered 56.73% of gene repertoire of *O. longistaminata* were identified (Figure 3e and Figure S6), indicating a highly conserved collinearity between the two *Oryza* species.

## 4 CONCLUSIONS

We report and annotate a high‐quality reference genome of *O. longistaminata* to enlarge the existing database of rice genome resources. A large collection of genomic sequences of Asian cultivated rice and its wild relatives has indisputably formed a solid base for hunting novel gene sources from the gene pool of wild rice germplasms. Thus, it is our hope that the generation of high‐quality reference genome of this African wild rice will enhance studies on comparative genomics of the genus *Oryza*, genomic basis underlying highly valued phenotypic traits, such as resistance, rhizomatousness and self‐incompatibility, and germplasm exploitation of useful genes in *O. longistaminata* for the future rice breeding programs.

## CONFLICT OF INTEREST DISCLOSURE

The authors declare that there are no conflicts of interest related to this manuscript.

## Supporting information

Supplemental Figure S1. The 17‐mer distribution of sequencing reads from *O. longistaminata*.
Supplemental Figure S2. Reads length distribution of the PacBio data for *O. longistaminata*.
Supplemental Figure S3. Workflow schema of the *O. longistaminata* genome project.
Supplemental Figure S4. Length distribution of scaffolds for the *O. longistaminata* genome. (a) Number of scaffolds in each of the indicated size ranges; (b) Percentage of occupied length in each of the indicated size ranges.
Supplemental Figure S5. Functional enrichment analysis of the *O. longistaminata* specific gene families.
Supplemental Figure S6. Collinear blocks between rice and *O. longistaminata*.
Supplemental Table S1. Summary of sequencing data to assemble the *O. longistaminata* genome.
Supplemental Table S2. Statistic of the *O. longistaminata* genome assembly using Illumina and PacBio sequencing reads.
Supplemental Table S3. Genome size of *O. longistaminata* estimated by flow cytometry and *k*‐mer analysis. *O. sativa* (the estimated genome size = 389 Mb) was employed as a inner standard, and the conversion factor used was 1 pg = 978 Mb (Doležel *et al*. 2003).
Supplemental Table S4. Statistic of the *O. longistaminata* genome assembly using Illumina, PacBio and Hi‐C sequencing reads.
Supplemental Table S5. Assembly statistics for the *O. longistaminata* genome sequences.
Supplemental Table S6. Validation of the *O. longistaminata* genome assembled using Illumina and PacBio sequencing reads through reads mapping, sequence alignments and BUSCO.
Supplemental Table S7. RNA‐Seq data of *O. longistaminata* from the four tissues using the Illumina Hiseq2000 platform.
Supplemental Table S8. Assembly of the *O. longistaminata* transcriptome using the Illumina Hiseq2000 platform.
Supplemental Table S9. Prediction of protein‐coding genes in the *O. longistaminata* genome assembled using Illumina and PacBio sequencing reads.
Supplemental Table S10. Summary of non‐coding RNA genes in the *O. longistaminata* genome assembly.
Supplemental Table S11. Statistics of repeat sequences in the *O. longistaminata* genome.
Supplemental Table S12. Summary of types and number of simple sequence repeats in the *O. longistaminata* genome.
