# Supplementary material for: Improved hybrid de novo genome assembly and annotation of African wild rice, Oryza longistaminata, from Illumina and PacBio sequencing reads
Source: Plant Genome. 2020 Mar 20;13(1):e20001. doi: 10.1002/tpg2.20001 (PMC12807249; doi:10.1002/tpg2.20001)
Supplement: Supplementary file 1 [file TPG2-13-e20001-s001.docx]

# Supplemental Figures

## Supplemental Figure S1. The 17-mer distribution of sequencing reads from *O.* *longistaminata*.

## Supplemental Figure S2. Reads length distribution of the PacBio data for *O. longistaminata*.

## Supplemental Figure S3. Workflow schema of the *O. longistaminata* genome project.

Supplemental Figure S4. Length distribution of scaffolds for the *O. longistaminata* genome**. (a)** Number of scaffolds in each of the indicated size ranges; **(b)** Percentage of occupied length in each of the indicated size ranges.

## Supplemental Figure S5. Functional enrichment analysis of the *O. longistaminata* specific gene families.

**Supplemental Figure S6. Collinear blocks between rice and *O. longistaminata*.**

# Supplemental Tables

## Supplemental Table S1. Summary of sequencing data to assemble the *O. longistaminata* genome.

| **Library** | **Platform** | **Insert size (bp)** | **Read length (bp)** | **Clean (Gb)** | **Coverage (×)*** |
| --- | --- | --- | --- | --- | --- |
| PE01 | Hiseq2000 | 300 | 100-120 | 15.09 | 38.49 |
| PE02 | Hiseq2000 | 360 | 100-120 | 44.52 | 113.57 |
| PE03 | Hiseq2000 | 500 | 100-120 | 7.96 | 20.31 |
| MP01 | Hiseq2000 | 2,000 | 35-100 | 41.20 | 105.10 |
| MP02 | Hiseq2000 | 4,000 | 40 | 1.70 | 4.34 |
| MP03 | Hiseq2000 | 5,000 | 100 | 21.49 | 54.82 |
| MP04 | Hiseq2000 | 6,000 | 40 | 2.43 | 6.20 |
| MP05 | Hiseq2000 | 8,000 | 70 | 31.86 | 81.28 |
| PB01 | PacBio | 20,000 | 8,324  (on average) | 7.28 | 18.57 |
| **Total** | | | | 173.53 | 442.68 |

* The estimated genome size: ~392Mb.

## Supplemental Table S2. Statistic of the *O. longistaminata* genome assembly using Illumina and PacBio sequencing reads.

| **Assembly features** | **Illumina only** | | **Illumina + PacBio** | |
| --- | --- | --- | --- | --- |
|  | **Contig** | **Scaffold** | **Contig** | **Scaffold** |
| **Number (#)** | 31,217 | 1,571 | 17,241 | 1,562 |
| **Total length (bp)** | 297,216,632 | 331,308,170 | 362,807,145 | 371,287,720 |
| **N50 (bp)** | 17,061 | 1,106,405 | 45,646 | 1,238,184 |
| **N90 (bp)** | 5,110 | 326,147 | 13,475 | 330,589 |
| **Largest (bp)** | 112,564 | 4,952,752 | 308,377 | 5,521,717 |
| **GC content (%)*** | 41.07 | 41.07 | 41.91 | 41.91 |

* GC content of the genome without N.

Supplemental Table S3. Genome size of *O. longistaminata* estimated by flow cytometry and *k*-mer analysis. *O. sativa* (the estimated genome size = 389 Mb) was employed as a inner standard, and the conversion factor used was 1 pg = 978 Mb (Doležel *et al*. 2003).

| **Species** | **Estimate Size (Mb)** | | | |
| --- | --- | --- | --- | --- |
|  | **Flow Cytometry Estimate (Mb)** | **17 *K*-mer Estimated Length (Mb)** | **Previously Estimated Size** |  |
| ***O. longistaminata*** | 362.90 | 392.00 | 389 ^a^ |  |

^a^ Uozu, Ikehashi *et al*. 1997;

## Supplemental Table S4. Statistic of the *O. longistaminata* genome assembly using Illumina, PacBio and Hi-C sequencing reads.

| **Features** | |
| --- | --- |
| **Estimated genome size (Mb)** | 392.00 |
| **No. of scaffolds** | 107 |
| **Total length of scaffolds (bp)** | 363,521,819 |
| **Scaffold N50** | 6,832,378 |
| **Scaffold N90** | 1,809,380 |
| **Longest scaffold (bp)** | 17,842,742 |
| **No. of contigs** | 15,883 |
| **Total length of contigs (bp)** | 355,146,980 |
| **Contig N50** | 46,493 |
| **Contig N90** | 14,202 |
| **Longest contig (bp)** | 308,377 |
| **Coverage of the assembled genome (%)** | 90.60 |
| **GC content (%)** | 41.90 |
| **No. of gaps** | 15,776 |
| **Total length of gaps (bp)** | 8,374,839 |
| **Longest gap (bp)** | 7,971 |

## Supplemental Table S5. Assembly statistics for the *O. longistaminata* genome sequences.

| **Chromosome ID** | **Contig number** | **Contig N50 (bp)** | **Chromosome**  **length (bp)** | **Nip Chromosome length (bp)** |
| --- | --- | --- | --- | --- |
| **1** | 2,048 | 49,297 | 47,172,414 | 43,270,923 |
| **2** | 1,865 | 46,491 | 47,119,111 | 35,937,250 |
| **3** | 1,650 | 50,520 | 40,086,801 | 36,413,819 |
| **4** | 1,392 | 47,076 | 33,924,873 | 35,502,694 |
| **5** | 1,263 | 44,492 | 26,615,265 | 29,958,434 |
| **6** | 1,666 | 47,907 | 38,458,780 | 31,248,787 |
| **7** | 1,411 | 47,525 | 28,005,666 | 29,697,621 |
| **8** | 1,049 | 43,114 | 24,070,047 | 28,443,022 |
| **9** | 763 | 44,461 | 16,523,016 | 23,012,720 |
| **10** | 770 | 45,425 | 19,397,591 | 23,207,287 |
| **11** | 1,196 | 41,863 | 22,575,426 | 29,021,106 |
| **12** | 988 | 46,362 | 20,880,455 | 27,531,856 |
| **Unmapped** | 1,250 | 12,373 | 6,521,175 | 0 |
| **Total** | 17,311 | 45,454 | 371,350,620 | 373,245,519 |

## Supplemental Table S6. Validation of the *O. longistaminata* genome assembled using Illumina and PacBio sequencing reads through reads mapping, sequence alignments and BUSCO.

|  | **Total** | **Mapped** | **Rate (%)** |
| --- | --- | --- | --- |
| **Reads mapping** | | | |
| Short libraries | 237,014,266 | 204,976,926 | 86.48 |
| **BUSCOs from Embryophyta lineage*** | | | |
| Complete | 1,440 | 1,294 | 89.86 |
| Duplicated | 1,440 | 79 | 5.49 |
| Fragmented | 1,440 | 56 | 3.89 |
| Missing | 1,440 | 90 | 6.25 |
| **Transcriptome assembled in this study**** | | | |
| Transcripts | 237,324 | 143,778 | 60.58 |

* The 1,440 BUSCO conserved genes used were collected from Embryophyta lineage.
** Hits with Coverage >= 90% and Identity >= 90%.

## Supplemental Table S7. RNA-Seq data of *O. longistaminata* from the four tissues using the Illumina Hiseq2000 platform.

| **Tissues** | **Read Length (bp)** | **Number of Pair Reads*** | **Clean Data (Gb)** |
| --- | --- | --- | --- |
| **30-d-roots** | 100 | ~33 M | ~6.36 |
| **30-d-shoots** | 100 | ~41 M | ~7.82 |
| **Panicles at booting stage** | 100 | ~29 M | ~5.51 |
| **Flag leaves at booting stage** | 100 | ~37 M | ~7.04 |
| **Total** |  | ~140 M | ~26.73 |

* M indicates million.

## Supplemental Table S8. Assembly of the *O. longistaminata* transcriptome using the Illumina Hiseq2000 platform.

|  | **Transcriptome Features** | **Values** |
| --- | --- | --- |
| **Longest contigs per component** | **Number of transcripts** | 111,105 |
|  | **Total length (bp)** | 74,561,481 |
|  | **N10 (bp)** | 3,378 |
|  | **N20 (bp)** | 2,477 |
|  | **N30 (bp)** | 1,912 |
|  | **N40 (bp)** | 1,461 |
|  | **N50 (bp)** | 1,064 |
|  | **Average length (bp)** | 671 |
|  | **GC content (%)** | 47.59 |
| **Total contigs** | **Number of transcripts** | 237,324 |
|  | **Total length (bp)** | 259,018,538 |
|  | **N10 (bp)** | 3,810 |
|  | **N20 (bp)** | 3,039 |
|  | **N30 (bp)** | 2,527 |
|  | **N40 (bp)** | 2,137 |
|  | **N50 (bp)** | 1,807 |
|  | **Average length (bp)** | 1,091 |
|  | **GC content (%)** | 47.46 |

Note: Transcriptome was assembled using Trinity (version 2.2.0) with default parameters.

## Supplemental Table S9. Prediction of protein-coding genes in the *O. longistaminata* genome assembled using Illumina and PacBio sequencing reads.

| **Gene Features** | **Statistic** |
| --- | --- |
| **Total number of protein-coding genes (#)** | 35,440 |
| **Total number of gene models (#)** | 39,278 |
| **Gene length in genome (%)** | 25.87 |
| **Average gene length (bp)** | 2,990 |
| **Gene density (gene per Mb)** | 95.45 |
| **Average CDS length (bp)** | 988 |
| **Average exon length (bp)** | 250 |
| **Average exons per gene (#)** | 4.6 |
| **Average intron length (bp)** | 500 |

## Supplemental Table S10. Summary of non-coding RNA genes in the *O. longistaminata* genome assembly.

| **Type** | **Number** | **Average Length (bp)** | **Total Length (bp)** | **% of Genome** |
| --- | --- | --- | --- | --- |
| **tRNA** | 755 | 75.0 | 56,658 | 0.0153 |
| **rRNA (8S)** | 1 | 111 | 111 | 0.0000 |
| **rRNA (18S)** | 2 | 1,790 | 3,580 | 0.0010 |
| **rRNA (28S)** | 1 | 4,083 | 4,083 | 0.0011 |
| **SnoRNA** | 274 | 98.3 | 26,935 | 0.0072 |
| **snRNA** | 143 | 142.3 | 20,353 | 0.0055 |
| **miRNA** | 299 | 131.8 | 39,398 | 0.0098 |

## Supplemental Table S11. Statistics of repeat sequences in the *O. longistaminata* genome.

|  | | **Length (bp)** | **Percentage (%)** |
| --- | --- | --- | --- |
| **Transposable Elements** | | 122074565 | 33.09 |
| **DNA transposons** | | 35213524 | 9.49 |
|  | ***En-Spm*** | 6541882 | 1.76 |
|  | ***Harbinger*** | 5459993 | 1.47 |
|  | ***MuDR*** | 8897049 | 2.40 |
|  | ***TcMar-Stowaway*** | 7499002 | 2.02 |
|  | ***Tourist*** | 81595 | 0.02 |
|  | ***hAT*** | 1364083 | 0.37 |
|  | ***Helitron*** | 2133743 | 0.57 |
|  | **Other** | 3236177 | 0.87 |
| **RNA Transposons** | | 81968418 | 22.08 |
|  | **Non-LTR Retrotransposon** | 4786404 | 1.29 |
|  | **LINE** | 3139512 | 0.85 |
|  | **SINE** | 1646892 | 0.44 |
|  | **LTR Retrotransposon** | 77182014 | 20.79 |
|  | ***Copia*** | 7551859 | 2.03 |
|  | ***Gypsy*** | 22330011 | 6.01 |
|  | **Other*** | 47300144 | 12.74 |
| **Other Repeats** | | 4892623 | 1.52 |
|  | **Low_complexity** | 643231 | 0.17 |
|  | **Satellite** | 225096 | 0.06 |
|  | **Simple repeats** | 4544416 | 1.22 |
|  | **Unknown** | 248456 | 0.07 |

* Non-autonomous LTR retrotransposons.

## Supplemental Table S12. Summary of types and number of simple sequence repeats in the *O. longistaminata* genome.

| **Repeat type** | **Number** | **Proportion**  **(%)** | **Total length (Kb)** | **Average length (bp)** |
| --- | --- | --- | --- | --- |
| **Mononucleotide** | 27,459 | 13.93 | 439.01 | 16 |
| **Dinucleotide** | 27,469 | 13.94 | 516.57 | 19 |
| **Trinucleotide** | 67,600 | 34.31 | 964.69 | 14 |
| **Tetranucleotide** | 50,857 | 25.81 | 675.80 | 13 |
| **Pentanucleotide** | 15,191 | 7.71 | 243.85 | 16 |
| **Hexanucleotide** | 8,477 | 4.30 | 166.70 | 20 |
| **Total** | **197,053** | **100.00** | **3,006.62** | **15** |
